# Avicenna and Cataracts: A New Analysis of Contributions to Diagnosis and Treatment from the Canon

**Published:** 2012-05-30

**Authors:** M Nejabat, B Maleki, M Nimrouzi, A Mahbodi, A Salehi

**Affiliations:** 1 Research Center for Traditional Medicine and History of Medicine, Shiraz University of Medical Sciences, Shiraz, Iran

**Keywords:** Avicenna, Cataract, Traditional medicine, Canon, History, Ophthalmology, Eye disorders

## Abstract

**Background:**

Physicians in ancient Persia played an important role in the development of medicine in the medieval era. One of the most influential figures of this era was Abu Ali Sina or Ibn Sina, known as Avicenna in the western world. The author of more than 200 books on medicine and philosophy, Avicenna followed and further expanded on the tradition of western philosophy and medicine introduced by Aristotle, Hippocrates and Galen. Few researchers have looked into the different medical issues in his best known work, the Canon of Medicine, particularly with regard to ophthalmology. In this analysis, Avicenna’s views on and contributions to the diagnosis and treatment of cataracts in his Canon were elucidated.

**Methods:**

We first reviewed an electronic copy of the Canon and then reviewed other important sources in traditional medicine including the Kamel-al-Sanaeh, Al-Havi (Continents) and Zakhireh-kharazmshahi, available in the Avicenna Special Traditional Medicine Library of Shiraz University of Medical Sciences. We also searched Medline, Embase, Scopus, Iranmedex and Science Iranian Database (SID) with these keywords: “traditional medicine,” “Avicenna,” “cataract”, “Canon”, “history”, “ophthalmology” and “eye disorders”.

**Results:**

According to the Canon, nozul-al-maa or cataract is an obstructive disease in which external moisture accumulates between the aqueous humor and the corneal membrane and prevents images from entering the eye. Avicenna classified cataracts on the basis of size, density and color. According to size, he identified two types of cataracts including complete and partial obstruction. According to the Canon, surgical intervention was necessary only for certain indications. Avicenna believed that opacity in the initial stages of cataract could be diminished by medicines and foods, and described several medicines for cataracts. He believed that surgery should be postponed until the liquid accumulation stopped, and the cataract reached its mature state. After surgery, according to Avicenna, the patient should avoid headache-inducing situations because headaches could lead to edema of the layers of the eye. He further emphasized that the patient’s psychological status played an important role in the success of surgery.

**Conclusion:**

An important aspect of Avicenna’s contribution to the medical management of cataracts was that he believed they could be cured by medication and nutrition in their early stages without the need for surgery. He also considered the patient’s mental status as an important factor contributing to the postoperative prognosis. Our review of Avicenna’s writings on eye disorders in the Canon of Medicine suggests that he had a rigorous approach to the diagnosis and management of patients suffering from eye disorders.

## Introduction

Physicians in ancient Persia played an important role in the development of medicine in the medieval era. One of the most influential figures in this era is Abu Ali Sina or Ibn Sina (980-1037 BC), known as Avicenna in the western world. Avicenna published more than 200 books on medicine and philosophy.[1] His masterpiece in medicine is the al-Qanun fi'l-tibb or the Canon of Medicine, which was completed in 1025.[2][3] This work presented a clearly organized summary of all medical knowledge of that time.[4][5] Originally written in Arabic, the book was later translated into a number of other languages including Persian, Latin, Chinese, Hebrew, German, French, and English.[6] Avicenna was one of the most eminent Muslim physicians and philosophers of his days, whose influence on Islamic and European medicine persisted for centuries.[7]

Avicenna followed and expanded on the tradition of western philosophy and medicine introduced by Aristotle, Hippocrates and Galen.[8] He created a system of medicine that is known as holistic medicine today.[9] His Canon contains some of the most illuminating ideas pertaining to the distinction between mediastinitis and pleurisy, the contagious nature of phthisis, and disease transmission through water and soil, in addition to careful descriptions of skin problems, sexual diseases and nervous ailments.[1] The Canon was translated into Latin by Gerard of Cremona in the 12th century. Its encyclopedic content, systematic arrangement and philosophical plan soon earned it a pre-eminent position in the medical literature of Europe, displacing the works of Galen and becoming the preferred textbook for medical education in schools throughout Europe, including the Universities of Montpellier (France) and Leuven (Belgium), as late as the year 1650.[1] The Canon retained its authority as a source of medical knowledge until the 18th century and early 19th century.[10][11] The influential Canadian physician Sir William Osler described the Canon as “the most famous medical textbook ever written”, noting that it remained “a medical bible for a longer time than any other work.”[12]

In recent times, researchers have taken a new look into different medical topics in the Canon. For example, different types of pulses[13] and trigeminal neuralgia[14] have been described in ways very similar to Avicenna's reports in his Canon. Savage referred to Avicenna in his discussion of surgical methods during the medieval era.[15] In the field of endocrinology, it is said that Avicenna was the first physician to describe thyroid-related orbitopathy[16] Musculoskeletal disorders in the Canon, such as rheumatologic diseases, have also been investigated.[17] Khodadoost argued that Iranian scientists including Avicenna played a major role in the development of ophthalmology in ancient Persia.[18] Madineh described bladder diseases in the Canon and compared Avicenna’s creative approach to the diagnosis and treatment of urologic disorders with conventional treatments.[19] Avicenna’s recommended treatment for tuberculosis (honey) has been the subject of some research in the field of respiratory tract infections.[20] Hatami explained the principles of clinical epidemiology in the Canon and Avicenna’s insights into the need for scientific experiments in medicine.[21] Avicenna’s innovative ideas on public health and preventive medicine have been the subject of other studies.[22][23] The Canon also devoted a chapter to the care of newborn infants, their hygiene, breastfeeding and upbringing.[7] In the field of neurosurgery, Avicenna provided extremely systematic knowledge on head traumas through his observations and experiences.[24]

**Fig. 1:**
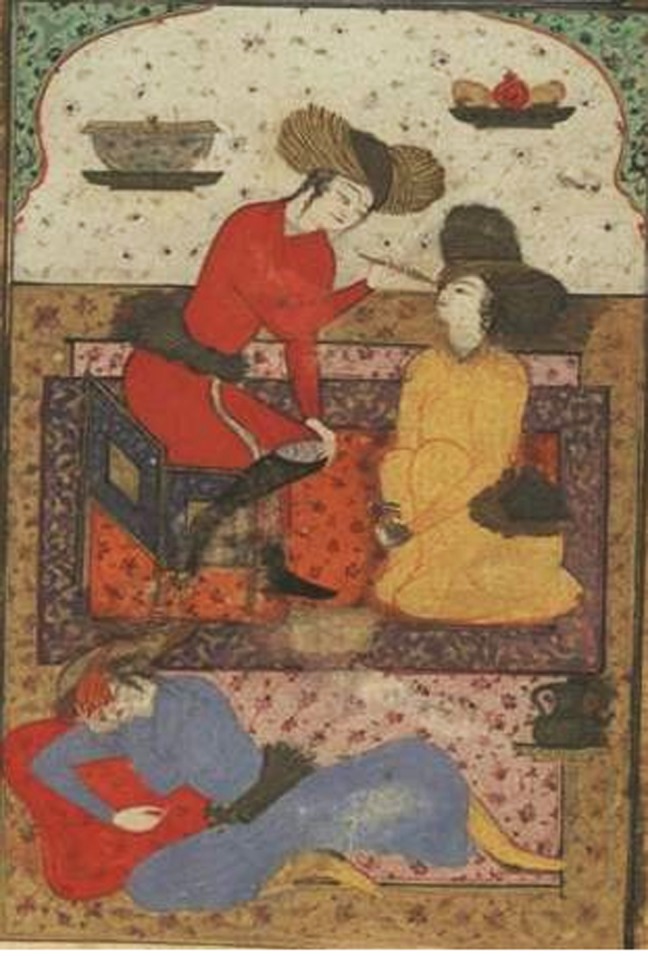
This picture of cataract surgery from the Resaleh fi-alein shows a patient during the operation above, and another patient below after surgery, with an eye dressing probably made of egg yolk violet oil (banafsaj).

**Fig. 2:**
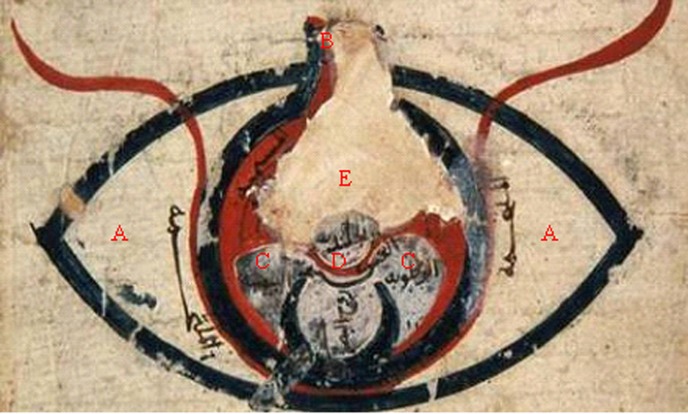
This illustration from the Resaleh fi-alein shows the normal anatomy of the eye according to Avicenna. A, Conjunctiva; B, Optic nerve; C, Aqueous in anterior chamber; D, Aqueous in posterior chamber E, Vitreous.

Despites these modern analyses, other areas of the Canon have received much less attention from modern historians. One such area is ophthalmology, which Avicenna covered in considerable detail in volumes one and three, presented detailed discussions of ophthalmic anatomy and diseases. Here we present Avicenna’s approach to the diagnosis and treatment of cataracts, with a special focus on the role of foods and medicines in the control of the early stages of cataract, and the methods of surgery to treat cataract in its mature states (Figure 1 and Figure 2).

## Materials and Methods

We first reviewed an electronic copy of the Canon and then examined important traditional Iranian medical sources such as the Majoosi-e-Ahwazi’s Kamel-al-Sanaeh, Rhazes’ Al-Havi (Continents) and Jorjani’s Zakhireh-kharazmshahi, all of which are held in the Avicenna Special Traditional Medicine Library of Shiraz University of Medical Sciences. We also searched Medline, Embase, Scopus, Iranmedex and Science Iranian Database (SID) with the related keywords “traditional medicine,” “Avicenna,” “cataract”, “Canon”, “history”, “ophthalmology” and “eye disorders”.

## Results and Discussion 

### Definition of cataracts in the Canon

Galen, the Roman physician who considered the lens to be essential to vision, believed that cataracts were thickenings of the humor on the surface of the lens. He proposed that “the dark retina lined the posterior surface of the lens, thus forming a mirror from which objects were reflected and on which impressions were registered and transmitted by the visual spirit through the hypothetic hollow optic nerves to the brain.”[25]

The name “crystalline” was first mentioned by Celsus (25 BC- 50 AD). The Canon was influenced by the writings of both Galen and Hippocrates. As a result, Avicenna’s definition of cataracts was very similar to the definition offered by Galen. The latter paid particular attention to the crystalline lens, which he described as a round lens in the middle of the eye. He concluded, “The crystalline lens was the principal instrument of vision which lied between the crystalline humor and the cornea and interfered with vision.”[25] Avicenna wrote, “Nozul-al-maa or cataract is an obstructive disease in which external (gharibeh) moisture accumulates between the aqueous humor and the corneal membrane at the pupil and prevents images from entering the eye”. By “external moisture” Avicenna apparently meant moisture that did not originate from the body and cannot be absorbed by body tissues and organs, and so accumulated as a source of infection or organ dysfunction.

### Grading of cataracts

Avicenna classified cataracts on the basis of their size, density and color. According to size, he distinguished two types of cataracts: Complete obstruction, in which the opacity covered the entire pupil, and partial obstruction, in which the opacity covered part of the pupil. He also defined two types of densities of cataracts: Thin, in which light was not blocked completely, and dense, in which the light was completely blocked. Avicenna also mentioned ten different colors of the opacity. According to the Canon, some types of cataracts were treatable by surgery (ghadh) with a specific instrument (a meghdahah), whereas others were resistant to surgical intervention. He noted that the bright colors responded well whereas the dark colors responded poorly to ghadh. Dense cataracts, which were hard and solid, were considered untreatable. Although his classification seems very simple in comparison with modern classifications, it was accurate enough for his major goal, i.e. to determine the method of treatment.[2]

### Symptoms of cataracts

Avicenna mentioned the following symptoms of cataracts: seeing small floating objects of fixed size and position, blurred vision, especially in only one eye, and doubled vision of shiny objects.[2]

### Treatments

Medication and nutrition

Avicenna believed that opacity could be diminished in the initial stages without surgery, and with medicines and foods only. As an example of this approach, Avicenna described a man who was treated by warding off inappropriate humors (istifragh), abstinence (hemiyah), decreasing his food intake, and abstaining from porridge (amraagh) and moist foods (morattibat). The patient was advised to eat only roasted meat (mashviyyat) and fried meat (ghalaya), and was given topical medicines to dilute (molattif) external moisture and corrode it (mohallil). His sight was successfully restored with this treatment. Avicenna added, “The person who has cataracts should avoid fullness and drinking and sexual intercourse. He should eat just one meal (lunch) per day and must avoid eating fish and fruit and heavy meat.”[2]

Avicenna also introduced several medicines for cataracts in the Canon which are potential research topics for ophthalmologists and pharmacologists. He noted that fennel juice mixed with honey and wild marjoram is effective in treating the initial stages of cataract.[2] Rhazes also mentioned in Al-Havi that wild marjoram was beneficial in the treatment of cataract. He maintained that corn cockle (Agrostemma githago), when mixed with orris root oil, is helpful to treat the initial stages of cataracts.[26] Orris contains flavenoids and isoflavones. It has been studied that herbal medicines containing flavenoids may slow down progression of cataracts in rats.[27][28] Javadzadeh et al. found that selenite-induced cataract in rats can be prevented by an eyewash prepared form onion juice which contains high content of flavenoids.[27] Another study by Goodarzi et al. showed that the consumption of some flavenoids such as quercetin reduced the accumulation of sorbitol in the lens of diabetic rats, and as a result slowed cataract progression. Tectorigenin and irigenin, two other isoflavone compounds, had similar results.[28] In addition to the Canon, many cataracts remedies were compiled in traditional Persian sources such as the Eksir-e-aazam, Gharabadin-e-kabir, Moalejat-e-Aghili and many other books, which are intriguing potential sources for further research.[29][30] Their authors were followers of Avicenna who explained his views in detail.

### Cataract surgery

Avicenna believed that certain conditions must be met to make a patient eligible for surgery. He mentioned that operations for pearl white, grayish white and turquoise white cataracts would result in good outcomes, whereas operating on chalky, green, dark and dense black cataracts would have a dismal prognosis. He also proposed an examination to determine whether the patient was appropriate for surgery: “If you examine the patient under half-shadow (shadow beside light) and press your finger gently on the eye, the cataract moves quickly under pressure and returns as soon as you remove your finger. Do not conduct this examination frequently because it may detach the opacity and make surgery very difficult.”[2]

### Patient selection 

According to Avicenna’s Canon of Medicine, the disease was the result of an imbalance in the four humors, and if a cataract was not due to trauma, it must be the result of humor imbalance and the accumulation of bad humors in the body generally and in the eyes locally. Treating patients without consideration of this fact almost ensured recurrence of the disease, according to him. Accordingly, the body should be cleansed of bad humors (phlegm and black bile). This could be facilitated by using medications such as ayarejat (purgatives which ward off bad humors via the bowels), and considering the patient’s status, the physician may decide to use fasd (a kind of phlebotomy performed with a special scalpel to open the appropriate vein and allow bad humors to exit the body). Regarding the cause of cataracts, Avicenna maintained, “If the cataract develops due to a fall or brain injuries, it is very difficult to cure it by surgery.”[2]

### Preoperative surveillance 

The patient who is a candidate for surgery must not undergo fasd just before the procedure. Avicenna believed that surgery should be postponed until fluid collection had stopped and the cataract had reached maturity, and recommended that the surgeon should wait until the opacity reached an appropriate density (neither too dilute nor too dense). Eating pea porridge (nokhodab) was recommended to protect the site where the surgical instrument penetrates the eye inferiorly. The patient who is a candidate for surgery should start eating fresh fish, moist foods, tonics and foods which will help revive him after the procedure. After these preoperative measures, ghadh can be considered for the patient.[2]

### Operative technique 

According to the Canon, the cataract operation should not take place in a very bright room or in front of a window. The patient must focus his gaze toward his medial canthus or his nose. The surgeon should insert the surgical instrument (meghdaha) from the limbus into the anterior chamber and proceed toward the pupil. He should then mobilize the visible opacities with the tip of the surgical instrument and push them to the lower angle of the anterior chamber until the patient’s vision becomes clear. He should use the surgical instrument to hold the opacities in their new position until they become stable. If the opacities move again after the surgical instrument is withdrawn, the surgeon should repeat the steps described above.

In surgical methods commonly used for cataracts in Avicenna’s era, the lens opacities were collected in the lower angle of the anterior chamber and surgeons did not remove them from the eye. If they had wished to do so, they would have had to make a larger incision in the eyeball, and this would have increased the risk surgical complications.[2]

### Postoperative measures 

After ghadh, the patient was instructed to avoid situations that could cause headaches, because this could lead to edema of the eye layers. Acute rage and coughing could result in early recurrence. The patient was advised to avoid drinking, sexual intercourse and bathing. After the operation, the eye was immediately covered with a piece of cotton, which was smeared with a mixture of egg yolk and violet oil (banafsaj). The intact eye was also covered to prevent eye movements. The patient was advised to lie on his back in a dark place for at least 3 days until he felt no discomfort. The bandage was changed every 3 days. The patient’s mental and psychological status played an important role in the success of surgery.[2]

## Conclusions and Suggestions for Further Research

Although the procedure and instruments used by Avicenna seem primitive in comparison to modern methods and equipment, they were considered effective and innovative one thousand years ago.[31] An important aspect of Avicenna’s views about cataracts is that he emphasized the treatment of cataracts in its early stages through medication and nutrition without the need for surgery. He also considered the role of the patient’s mental and psychological status in the success of surgery. These elements of patient care are potentially worthwhile topics for further research. Our review of the section on the treatment of eye disorders in the Canon of Medicine reveals that Avicenna had a meticulous approach to diagnosis and management for patients who suffered from eye disorders. Ultimately, his decision regarding the choice of the most appropriate method of treatment was based on precise clinical observation and examination.[2]
